# Morphostructural Characterization of Hunting Dog Packs (Rehalas) Using Multivariate Methodology

**DOI:** 10.3390/ani15192908

**Published:** 2025-10-06

**Authors:** Carlos Poderoso Martínez, Ana González-Martínez, Manuel Luque Cuesta, Evangelina Rodero Serrano

**Affiliations:** 1 Doctoral Program in Natural Resources and Sustainable Management, University of Córdoba, 14071 Córdoba, Spain; poderoso967@gmail.com; 2 Animal Production Department, University of Córdoba, 14071 Córdoba, Spain; erodero@uco.es; 3 Royal Spanish Federation of Livestock Purebred Associations (RFEAGAS), Castelló, 45, 28001 Madrid, Spain; manuel.luque@rfeagas.es

**Keywords:** canine, morphology, morphometric measurements, discriminant analysis, Spanish breeds

## Abstract

**Simple Summary:**

This investigation on hunting dog packs from the Sierra Morena in Córdoba aimed to deepen our knowledge of the dogs’ morphological characterization. The results showed that the Large-size Podenco Andaluz is the most predominant type of dog in the study area. This dog exhibits clear sexual dimorphism, and breeders choose it for its versatility during long hunting days. Morphologically, it is distinct from other Spanish dog breeds used in the Spanish traditional big game hunt known as Montería. This study provides an update on the morphostructural characteristics of the Large-size Podenco Andaluz, as the last study was conducted four decades ago.

**Abstract:**

On the south–central Iberian Peninsula, big game hunting is traditionally carried out using big-game hunting under the “Montería” modality, with dog packs. Breeders of these dogs value their versatility in both chasing and capturing prey. In this context, the most popular breed is the Large-sized Podenco Andaluz, colloquially known as Podenco Campanero. In this study, we aimed to morphologically characterize the hounds of the Sierra Morena in Córdoba and evaluate their possible relationships with other Spanish hunting dog breeds. For this purpose, 255 dogs were measured to obtain sixteen morphometric measurements and eleven indices. To assess morphostructural differentiation, we applied multivariate methodologies. The Podenco Campanero exhibited pronounced sexual dimorphism, with males being significantly (*p* < 0.001) longer, taller, wider, and deeper than females. The morphostructural model of this breed demonstrated considerable homogeneity and harmony, and the population exhibited distinct morphostructural characteristics, with body size and regional width varying between individuals. The morphometric characteristics of the breeds used in Monterías on the central and southern Iberian Peninsula highlight that the diversity of these local genetic resources is shaped by genetic relationships and selective breeding models chosen by dog pack breeders, which depend on the hunting modality and the terrain characteristics where it is practiced.

## 1. Introduction

The domestication of dogs (*Canis lupus familiaris*) approximately 15,000 years ago enabled humans to selectively breed animals adapted to diverse functions, resulting in the extraordinary morphological variability observed today [1,2,3].

One of the oldest and most enduring human–dog relationships is their collaboration in hunting, which has driven the development of specialized hunting breeds [4,5,6,7,8]. In Spain, this collaborative hunting method is called “Montería,” which is a millennia-old tradition officially regulated in 1180 AD [9]. In Portugal, an equivalent practice is known as “Montaria” [10]. Similar practices exist in other countries, such as “deer hunting” in the United Kingdom and “chasse à courre” in France. Italy also has comparable traditions [11,12].

Traditionally, a dog pack used to hunt wild ungulates and boars in Spain is known as a “rehala” (hunting dog pack) and usually consists of 10 to 12 pairs of dogs (known as “colleras”) led by a “rehalero” or “perrero” [9]. These canine teams are primarily trained for driven hunts of deer and wild boar. The performance of the hunting dog pack earns its breeder (rehalero) prestige at least equal to that of a successful shooter [13]. The type of dog is chosen because it shares certain physical (e.g., size, weight, and high biting force) and social (e.g., hunting in packs) characteristics with Iberian wolves [14]. The composition of the dog pack is also influenced by the dominant game species in each hunting area; many hunters prefer hounds when deer are abundant, while others opt for mastiff crossbreeds for wild boar hunting.

In Spain, the millennia-old tradition of “Montería” relies on organized hunting dog packs (“rehalas”) typically composed of Podencos, Mastiffs, and Alanos [9,15]. Beyond Spain’s borders, dog breeds used to hunt deer and wild boar include the Grand Gascon Saintongeois in France [16]; the Ariege Hound, Istrian Hound, and Italian Hound in Italy [12]; and the Drahthaar in Germany [16].

Despite the existence of well-defined hunting dog breeds in Spain, it is common to find hunting dog packs composed of mixed-breed dogs or selectively bred populations that, despite being phenotypically homogeneous and easily recognizable, have not yet been officially recognized as breeds.

The choice of dog pack is primarily influenced by the terrain’s topography and the predominant game species, but the preferences of each breeder also play a role. In the Sierra Morena region, the predominant breed is the Large-sized Podenco Andaluz (RSCE Nº 401), which has rough hair and is locally known as Podenco Campanero. This breed is valued for its ability to work in rough terrain and confront big game such as deer and wild boar [17,18,19,20,21]. However, despite their phenotypic homogeneity and functional importance, many of these dogs are not officially recognized as breeds. Selection is often carried out locally by breeders, leading to considerable intrabreed variability in morphological and coat traits.

The Food and Agriculture Organization of the United Nations (FAO) recommends phenotypic characterization as the first step in defining animal genetic resources [22]. The methodology employed involves a descriptive analysis of morphological and coat characteristics. Regarding morphostructural traits, morphometric data are obtained and analyzed using multivariate methods such as Principal Component Analysis (PCA) to identify the most significant variables in breed characterization. This is complemented by Canonical Discriminant Analysis to examine the degree of differentiation between populations [23,24,25,26,27,28,29,30,31]. This methodology is advantageous due to its ease of application and low cost, making it particularly useful for developing countries. Such studies have been applied to characterize and differentiate Spanish canine populations [32] and to evaluate the influence of morphostructure on dog function [33].

The Sierra Morena hunting area in the province of Córdoba covers an area of 437,852 ha and is predominantly forested land with different uses: grazing areas, pastures, dense trees, and scrubland [34]. Deer are the main big game hunted in this mountain area, followed by wild boar.

The objective of this study was to morphostructurally characterize the dogs that comprise hunting dog packs in the Sierra Morena region of Córdoba and evaluate their possible relationships with other Spanish hunting dog breeds. A detailed morphostructural characterization of these dogs is essential to safeguard their genetic diversity, support their potential recognition as distinct breeds, and ensure their continued functional role in one of Spain’s most important hunting traditions.

## 2. Materials and Methods

### 2.1. Data Collection

**Figure 1:**
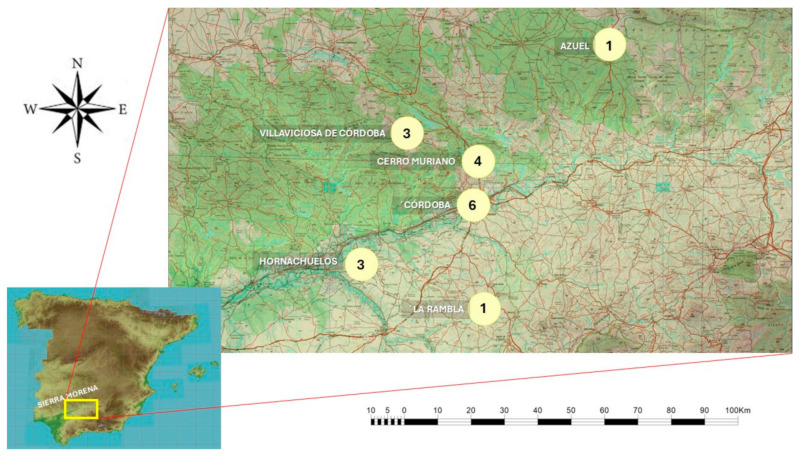
Geographic location and number of sampling dog packs. Source: Author’s own.

For the characterization of dogs in the Sierra Morena region of Córdoba, data were collected between 2022 and 2023 from a sample of 18 breeders (“rehaleros”) located in the surroundings of Sierra Morena, Córdoba, Spain (Figure 1). This region covers approximately 339,000 ha in the central–northern part of the province. Its climate is classified as Mediterranean subtropical, with average temperatures ranging from 15 to 20 °C and annual precipitation between 600 and 890 mm. The vegetation consists of Mediterranean scrubland, featuring various Quercus species (holm oaks, cork oaks, and gall oaks), and low scrubland [35].

To organize sample collection and visits, voluntary collaboration was requested of the breeders through the Association of Dog Packs of Córdoba. All morphometric measurements and other phenotypic data were collected by the same person to ensure consistency.

From the interviews conducted, it was determined that the dog packs of Sierra Morena, Córdoba, predominantly consist of the Large-sized Podenco Andaluz breed, also known as Podenco Campanero, while specimens of other breeds were rare (Table 1).

**Figure 2:**
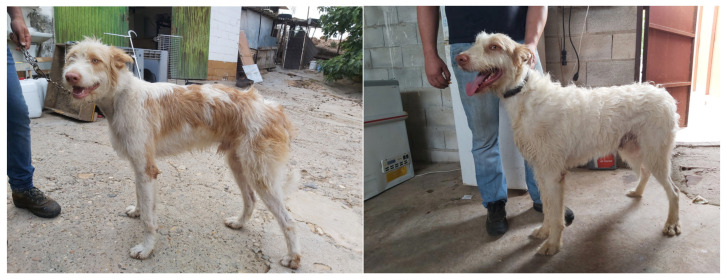
Male and female Large-sized Podenco Andaluz dogs. Source: Author’s own.

Based on these findings, we focused on the Large-sized Podenco Andaluz (Podenco Campanero), as it is the most representative breed in the Montería hunting system in Sierra Morena, Córdoba (Figure 2). A total of 255 Podenco Campanero dogs were sampled (173 males and 82 females). The average (±standard error) size of the dog packs was 51.87 ± 3.49 dogs [21], with approximately 10–15 breeding dogs per pack, selected based on their superior morphological and genetic qualities, according to the dog owners’ criteria.

To assess the degree of differentiation between the Podenco Campanero and other native Spanish hunting dog breeds used in Montería, the following comparative samples were included: Large-sized Podenco Andaluz (154 males and 75 females), Valdueza (81 males and 22 females), Spanish Greyhound (44 males and 42 females), Spanish Mastiff (34 males and 34 females), and Podenco Paternino (71 males and 74 females). A sample of Podenco Campanero and Spanish Mastiff crossbreeds from a dog pack reared in the Sierra Morena area of Córdoba was also included. The data collection for these breeds was based on that used in the study by González et al. [33]. However, in the case of the Valdueza breed, the samples were collected between 2017 and 2018 [36].

### 2.2. Morphometric Variables

The dogs were measured using a measuring stick, calipers, and a tape measure. A total of 16 body measurements were recorded, focusing on bony prominences to avoid the influence of body conformation (Table 2). Based on these measurements, 11 body indices were also calculated, following the methodology described by González et al. [32].

### 2.3. Statistical

The main objective of this study was to morphometrically describe the Podenco Campanero using descriptive statistics for 16 morphometric variables and 11 indices. Normality and homoscedasticity were verified using the Kolmogorov–Smirnov and Bartlett tests. Student’s *t*-tests were conducted to analyze the effect of sex on these measurements. Additionally, a Pearson correlation matrix was used to examine the relationships between the morphometric traits.

To evaluate the morphostructural differentiation of the Podenco Campanero from other native Spanish dog breeds, multivariate analysis of variance (MANOVA) and Tukey’s test were conducted. All morphometric traits were included except for the height of the substernal hollow, as this measurement was unavailable for some populations.

A discriminant analysis was performed to identify the variables with the highest discriminant power and to determine the accuracy of classification matrices in assigning dogs to their respective groups. Additionally, Mahalanobis distances between different groups were calculated and represented using a cluster tree. Canonical discriminant procedures were used to clarify the classification of the dogs into distinct populations, with the results displayed through a graphical representation of canonical coefficients.

The significance level for model inclusion and retention was set at *p* < 0.05. All statistical analyses were performed using Statistica for Windows 12.0 (StatSoft, Inc., Tulsa, OK, USA).

## 3. Results

### 3.1. Morphometric Characterization of Large-Sized Podenco Andaluz

The Large-sized Podenco Andaluz or Podenco Campanero exhibited marked sexual dimorphism, with males being significantly (*p* < 0.001) longer, taller, wider, and deeper than females (Table 3). This breed showed a greater height at the anterior third compared to the posterior third (64.07 ± 0.29 cm vs. 63.64 ± 0.27 cm), as well as elongated proportions, since the body length exceeded the height at the withers (67.34 ± 0.28 cm vs. 64.07 ± 0.29 cm). The head had a length of 13.26 ± 0.07 cm and a width of 12.29 ± 0.07 cm.

At the thoracic level, the Podenco Campanero presented a width, depth, and perimeter of 16.55 ± 0.09 cm, 26.67 ± 0.10 cm, and 75.83 ± 0.23 cm, respectively. At the rump level, length and width showed little variation, with values of 17.57 ± 0.13 cm and 17.24 ± 0.14 cm, respectively. Lastly, the forelimbs had a perimeter one centimeter greater than the hindlimbs (12.98 ± 0.06 cm vs. 11.93 ± 0.06 cm).

The Podenco Campanero is a dolichocephalic breed (CI = 48.35%), with significant body development (BI = 88.89%) and a square body shape at both the general body (PrI = 95.28%) and rump level (PI = 98.30%). Males showed significantly higher values (*p* < 0.001) for the cephalic index, body index, and dactylo-costal index, whereas females exhibited a higher value in the relative proportionality of the thoracic index (*p* < 0.05) (Student’s *t*-test).

The Podenco Campanero’s degree of homogeneity is moderate to high, as the coefficients of variation showed values below 10% except for the rump, which presented values between 10% and 12%.

Pearson correlation coefficients were higher in males than in females, with 71.67% and 57.50% of them being significant (*p* < 0.05), respectively (Table 4). In males, HaW showed a highly significant (*p* < 0.05) positive correlation with HR (r = 0.90), BL (r = 0.58), and HL (r = 0.57), whereas in females, HaW was only correlated with HR (r = 0.95). In both sexes, HL showed a highly significant (*p* < 0.05) positive correlation with SL (r = 0.87 in males; r = 0.90 in females) and FL (r = 0.69 in males; r = 0.84 in females), as well as RW with RL (r = 0.72 in males; r = 0.71 in females) and FC with HC (r = 0.62 in males; r = 0.76 in females). Furthermore, in males, BL showed a highly significant (*p* < 0.05) positive correlation with HR (r = 0.61), while TC was correlated with DsD (r = 0.58), HL (r = 0.52), and SL (r = 0.51). In females, SW was correlated with ChW (r = 0.62) and SC (r = 0.55).

### 3.2. Application of Discriminant Analysis for Breed Differentiation

Table 5 presents the morphostructural characteristics of five Spanish dog breeds and the population resulting from the crossbreeding of the Podenco Campanero and the Spanish Mastiff. The MANOVA analysis for the six dog populations (Podenco Campanero, Podenco Paternino, Valdueza, Spanish Greyhound, Spanish Mastiff, and the Podenco Campanero × Spanish Mastiff crossbreed) showed a highly significant effect (*p* < 0.05) of both breed and sex on all morphometric traits except for SW in both breed and sex and RW in sex. However, the interaction between breed and sex was only significant (*p* < 0.05) for TC and FC. Each population exhibited distinct morphostructural characteristics, with differences in body and regional size and width. Generally, the Spanish Mastiff was the tallest, longest, and deepest of the canine populations studied. In contrast, the Podenco Paternino showed the lowest values for height, length, and depth except for head, shoulder, and rump width, which were lower in the Spanish Greyhound.

The discriminant function, obtained with fifteen measurements, showed that thirteen (86.67%) variables in males and all (100.00%) in females were accepted in the model, with ten (76.92%) and all (100.00%) measurements being significant (*p* < 0.05), respectively (Table 6).

The relationships among the discriminant variables revealed a specific morphological pattern for each population, with a correct classification percentage of 100% in females and 96.24% in males (Table 7). Misclassification in Podenco Campanero individuals was observed only in males, with errors occurring between Valdueza and the Podenco Campanero × Spanish Mastiff crossbreed population.

**Figure 3:**
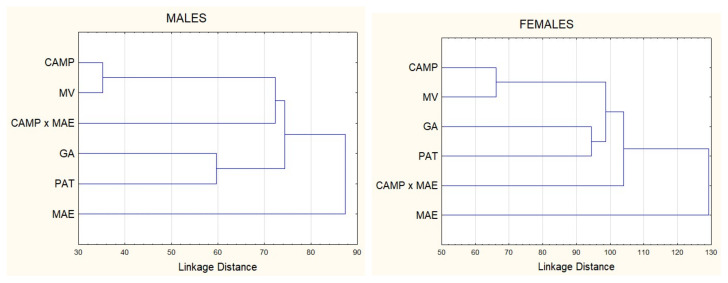
Mahalanobis distances for 399 males and 251 females of five Spanish dog breeds1 and one crossbreed population using fifteen morphometric variables. CAMP = *Podenco Campanero*; CAMP × MAE = *Podenco Campanero* × Spanish Mastiff; MV = *Valdueza*; GA = Spanish Greyhound; MAE = Spanish Mastiff; PAT = *Podenco Paternino*.

**Figure 4:**
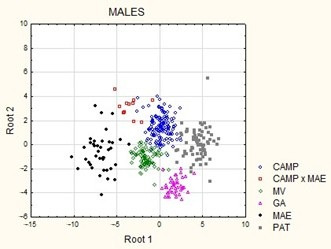

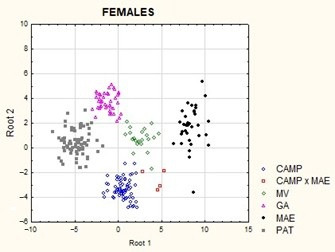
Canonical representation of 399 males and 251 females of five Spanish dog breeds1 and one crossbreed population using fifteen morphometric variables. CAMP = *Podenco Campanero*; CAMP × MAE = *Podenco Campanero* × Spanish Mastiff; MV = *Valdueza*; GA = Spanish Greyhound; MAE = Spanish Mastiff; PAT = *Podenco Paternino*.

Morphostructural differences among the six analyzed dog populations were visually represented through morphometric measurements in a graphical representation of Mahalanobis distances (Figure 3). The first cluster grouped individuals from the Podenco Campanero and Valdueza breeds, while the second cluster included individuals from the Spanish Greyhound and Podenco Paternino breeds. The crossbreed population (Podenco Campanero × Spanish Mastiff) was positioned between the first and second clusters in males, whereas in females, this population was more distant from both clusters. The Spanish Mastiff was the most distinct breed in both sexes. These results are consistent with the canonical analysis plot, in which the Podenco Campanero population was closer to the Valdueza breed and the Podenco Campanero × Spanish Mastiff crossbreed. This last breed was also positioned further in the plane compared with other dog populations (Figure 4).

## 4. Discussion

### 4.1. Types of Dogs Used in Monterías in Sierra Morena, Córdoba

The results indicate that rehaleros in Sierra Morena, Córdoba, primarily use the Large-sized Podenco Andaluz breed in traditional big game hunting, such as Montería, where they rely on their dogs to hunt ungulates and wild boars [21].

This breed, originally from the Sevillian municipality of La Campana, has the ability to endure and intercept wild ungulates before they can take refuge in dense mountain undergrowth. Additionally, they are brave and bold enough to stop the game (personal communication). The Podenco Andaluz is characterized as an excellent game finder, with a strong sense of smell, light build, high speed, and high enthusiasm, making it an ideal breed for locating and flushing out game. However, although some individuals are highly courageous, a dog pack composed solely of hounds would struggle to subdue a large wild boar. To ensure the hounds do not lose confidence and abandon the boar in favor of deer, these dog packs are complemented by a few gripping dogs that can assist in restraining a large wild boar if needed. As a result, many dog packs in southwestern Spain reinforce their hounds with one or two pairs of Alanos, Presa Canarios, Dogo Argentinos, etc. [17].

In rougher terrains, the Podenco Campanero is often crossbred with the Spanish Mastiff, as this combination seems to be the most effective for Montería in such environments. The crossbreed retains the scenting ability and agility of the Podenco while incorporating the endurance and bravery of the Mastiff, providing the strength and courage necessary to drive wild boars out of hiding. These dogs are also capable of hunting for several consecutive days and can endure harsh weather conditions, such as cold and rain, better than other hounds [16].

The Podenco Campanero is widespread across Andalusia, Extremadura, and central Spain, although the highest population density and purest specimens are found in the Andalusian provinces of Córdoba, Seville, and Jaen [37]. In other areas of the southwestern Iberian Peninsula, breeders rely on breeds such as the Podenco Paternino (in the Huelva mountains) or the Valdueza (in the hunting estates of Castilla-La Mancha and Extremadura). These breeds share a common ancestor: the Podenco Campanero.

### 4.2. Morphometric Characteristics of Podenco Campanero in Sierra Morena, Córdoba

The Podenco Campanero used in the dog packs of Sierra Morena, Córdoba, is a medium-sized dog (27 ± 6 kg), with proportions ranging from medium to stocky, and a white or cinnamon coat [38]. It exhibits significant sexual dimorphism, with females being smaller than males, which is consistent with previous descriptions of the breed [37]. Over the past forty years, its morphometric characteristics have changed, with the modern Podenco Campanero exhibiting a larger body size in terms of length, width, and depth (e.g., BL = 68.62 cm vs. 61.76 cm; RW = 17.60 cm vs. 12.46 cm; DsD = 26.67 cm vs. 24.27 cm). Conversely, skull length (12.21 cm vs. 14.38 cm) and rump length (17.57 cm vs. 20.39 cm) have decreased. The skull length variation may be due to differences in measurement reference points, particularly in locating the exact frontonasal suture site. Additionally, when comparing head sizes over four decades, males’ head sizes have remained nearly unchanged (25.73 cm vs. 25.35 cm), while females’ head sizes have increased (24.93 cm vs. 23.69 cm) [37]. The rump length also decreased by approximately 2 cm in both sexes, possibly as an adaptive response to the rugged terrain in which these dogs hunt in Sierra Morena, Córdoba [39].

The morpho-structural model of the Podenco Campanero in Sierra Morena, Córdoba, is highly harmonious, as evidenced by the high number of significant correlations between morphometric variables. This strong morphological balance may reflect an excellent selection process by breeders, who aim to create a fully functional hunting dog with uniformity among individuals within each dog pack. The high number of significant correlations may also be attributed to the morphological similarity of the sampled animals, as coefficients of variation below 10% indicate a medium-to-high level of homogeneity [40].

### 4.3. Differences Among Spanish Hunting Dog Breeds Used in Monterías

A previous study evaluated the morphological characteristics of various Spanish dog breeds and their classifications within FCI groups based on purpose, morphology, and work function [33]. This research includes three Spanish dog breeds traditionally used in big game hunting (Monterías) in southern Spain, as well as two breeds, the Spanish Mastiff and the Spanish Greyhound, which have frequently been crossbred with hunting dogs to enhance their functionality. A population resulting from the crossbreeding of the Large-sized Podenco Andaluz and the Spanish Mastiff from a dog pack in the study area was also included.

Among these breeds, the Spanish Mastiff exhibited the highest values in most morphometric measurements, followed by the Spanish Mastiff and Podenco Campanero crossbreed. The Mastiff, which has been used since ancient times to protect livestock against predators such as wolves, is a large, powerful, and muscular dog with a compact skeletal structure [41]. In contrast, the Podenco Campanero exhibits an intermediate morpho-structure, while the Podenco Paternino shows the lowest values in almost all the morphometric variables considered.

Crossing the Podenco Campanero with the Spanish Mastiff results in a dog with a broader chest and rump, providing the strength needed for wild boar hunting over long hunting days, which is characteristic of gripping breeds. However, the Valdueza, a population derived from previous efforts at this type of crossbreeding, has undergone careful selection, resulting in an intermediate morpho-structure rated between the Podenco Campanero and the Spanish Mastiff.

Multivariate analysis confirmed that the Podenco Campanero of Sierra Morena, Córdoba, has a unique morphological identity. Almost all morphometric variables considered play a key role in distinguishing the different canine breeds used in Monterías in Mediterranean forest ecosystems. The FAO recommends using discriminant analysis to validate breed differences based on morphological or morphostructural models [22].

The classification matrix also highlights possible similarities between breeds. Our results show that errors in assigning animals to their original population are very low and occur only in some males. All errors, except for one Valdueza individual classified as a Spanish Greyhound, occurred among the Podenco Campanero, Valdueza, and Podenco Campanero × Spanish Mastiff crossbreed. These results are consistent with the breed origin of the Valdueza, which is based on a cross between the Podenco Campanero and the Spanish Mastiff [17]. No classification errors were found among females, which may be due to breeders’ meticulous selection of future breeding females. Among males, only the highest-quality individuals were retained for breeding (a small number compared to females), while the rest joined the dog pack without reproducing, according to information gathered from interviews with breeders.

As expected, Mahalanobis distances reveal that the Podenco Campanero and Valdueza form a cluster. In addition to genetic origin, the identified morpho-structural models reflect the selective criteria used by breeders when choosing breeding stock, as well as the most functionally favorable adaptations for hunting in similar terrains. The cluster formed by the Podenco Paternino and the Spanish Greyhound includes the lightest and fastest breeds, which are preferred in more open Mediterranean hunting systems, such as the Sierra de Aracena and Picos de Aroche [42]. The Podenco Paternino exhibits the greatest morphological differences from the other studied breeds, as it is smaller than the Podenco Campanero, despite having a common origin. However, these dogs hunt in distinct regions.

The representation of the canonical coefficients shows individuals from each of the studied canine breeds within the same plane. Discriminant analysis positions the Spanish Mastiff apart from all other populations, perhaps due to its ancient selection for different purposes, such as guarding and protecting livestock against predators such as wolves.

Males resulting from the crossbreeding of the Podenco Campanero with the Spanish Mastiff were positioned closer to the Valdueza breed, while females were positioned between the Podenco Paternino and the Spanish Mastiff. This latter finding could be attributed to the smaller sample size of females. However, the sample was determined by the composition of the dog pack in Sierra Morena, Córdoba, which predominantly consists of the Podenco Campanero breed. Other breeds are present only anecdotally, either due to adherence to traditional practices or because no dog adapts better to the hunting environment than the Podenco Campanero. This does not exclude support from other specialized gripping breeds during long hunting days [17], but these dogs would never constitute the majority of the dog pack, nor would the owner maintain breeders from these populations; instead, they acquire these dogs from other breeders.

The proximity of Spanish Mastiff individuals to the population resulting from their crossbreeding with the Podenco Campanero is due to their genetic origin, as the crossbreed may not yet be fully established, unlike the Valdueza. The Valdueza is closer to the Podenco Campanero, possibly because decades of selection for desired traits have resulted in morphostructural characteristics more similar to those of the Podenco Campanero than the Spanish Mastiff. Regarding the Valdueza breed, it has been highlighted that “In this dog, a masterful fusion has been achieved, combining the power and presence of the Mastiff, the hardiness, endurance, agility, and alertness of the large, rough-haired Podenco Andaluz, and the barking and tracking abilities of the Griffon. This result has been reached through many generations of selection, establishing a very distinctive phenotype with the best temperament traits for pack hunting” [36].

The morphostructure of animals results from their origin, human selection, and adaptation to the environment. The canine populations considered in this study have been found to differ morphostructurally, with some being larger, broader, and deeper, while others are smaller and lighter.

The three dog pack populations included in this study (Podenco Campanero, Podenco Paternino, and Valdueza) present distinct morphostructures, with the Valdueza being the largest, the Podenco Paternino the smallest, and the Podenco Campanero falling between the two. These three breeds carry out their hunting activities in geographically distinct areas: Sierra Morena in Córdoba (Podenco Campanero), Sierra de Aracena y Picos de Aroche in Huelva (Podenco Paternino), and Montes de Toledo in Ciudad Real (Valdueza).

All three geographic areas share a Mediterranean-type climate and landscape characterized by an abundance of Quercus trees and low scrubland [35]. However, they differ in the predominant big game species, with deer being the most abundant in Córdoba and Ciudad Real, while wild boar dominate in Huelva [43]. Altitude is also a key factor in these regions: the Sierra Morena in Córdoba does not exceed 800 m in altitude [44], whereas the Sierra de Aracena y Picos de Aroche reaches 1040 m [43], and the Montes de Toledo rise to 1447 m [45].

Studies on goats have shown that the rump tends to shorten as altitude increases [39], which aligns with our findings. The Podenco Paternino, which hunts in higher-altitude terrains than the Podenco Campanero, has a shorter rump despite sharing the same genetic origin. However, the Valdueza, despite hunting in high-altitude areas, has a longer rump, likely due to its genetic origin. As previously mentioned, this breed results from the crossbreeding of the Podenco Campanero and the Spanish Mastiff, the latter being the breed with the longest rump.

The jaw, a region not considered in this study as it is not part of the animals’ morphostructure, plays an important role in the world of pack dogs, as it is crucial for gripping prey once taken down. The greater presence of wild boars in the mountains of Huelva may explain why the Podenco Paternino has a highly developed jaw, both skeletally and muscularly [19]. However, the Podenco Campanero and the Valdueza also exhibit well-developed jaws [38,46], as modern pack dog breeders seek versatile animals capable of both chasing and gripping prey.

## 5. Conclusions

The hunting dog packs of Sierra Morena, Córdoba, are primarily composed of Large-sized Podenco Andaluz or Podenco Campanero dogs, which are sometimes crossbred with the Valdueza or Spanish Mastiff to enhance grip strength and endurance.

The Podenco Campanero exhibits significant sexual dimorphism, with males being taller, wider, and deeper than females. Over the last three decades, its morphology has significantly adapted to its environment, increasing in size.

The morphometric characteristics of the canine breeds used in Montería in the central and southern Iberian Peninsula highlight that the diversity of these local genetic resources is determined by their genetic relationships and by the selective breeding models chosen by dog pack breeders, which depend on the hunting modality and the characteristics of the terrain on which it is practiced.

## Figures and Tables

**Table 1:** Distribution of dogs by breed and sex in the studied dog packs.

Breed ^1^	Absolute Frequency ^2^	Relative Frequency
CAMP	229 (154 M y 75 F)	89.80
CAMP × MAE	15 (11 M y 4 F)	5.88
Alano	4 (3 M y 1 F)	1.57
Dogo Argentino	3 (2 M y 1 F)	1.18
Agarre Cruce	1 (M)	0.39
Naveño	1 (F)	0.39
Anglo	1 (M)	0.39
Fox Terrier	1 (M)	0.39

^1^ CAMP = Large-sized *Podenco Andaluz*; CAMP × MAE = Large-sized *Podenco Andaluz* × Spanish Mastiff; ^2^ M = males; H = females.

**Table 2:** Body measurements and estimated body indices.

Trait	Abbreviation	Description
Distance, cm		
Head length	HL	distance from the nape to the cranial border of the snout
Height at withers	HaW	distance from the highest point of the processus spinalis of the first thoracic vertebra (T1 and T2) to the floor
Body length	BL	distance from the most cranial point of the sternum to the most caudal point of the pin bone
Rump length	RL	distance from hips (Tuber coxae) to pins (Tuber ischii)
Height at rump	HR	distance from the highest point of the rump (ilium) to the floor
Dorso-sternum diameter	DsD	distance from the lowest point in the wither decline to the sternal area
Skull length	SL	distance from the nape to the occipital crest
Face length	FL	distance from the occipital crest to the cranial border of the snout
Height of substernal hollow	HsH	distance from substernal hollow to the floor
Width measurements, cm		
Head width	HW	distance between two zygomatic arches
Rump width	RW	distance from the left to the right point of hip
Shoulder width	SW	distance from left to right upper arm (pars cranialis of the tuberculum majus humeri)
Chest width	ChW	distance from the left to the right point of the back
Perimeters, cm		
Thoracic circumference	TC	measured in place of the saddle girth
Forelimb circumference	FC	smallest circumference of tibia bone of the forelimb
Hindlimb circumference	HC	smallest circumference of tibia bone of the hindlimb
Index		
Cephalic index	CI	HW × 100/HL
Body index	BI	BL × 100/TC
Proportionality index	PrI	HaW × 100/BL
Thoracic index	TI	ChW × 100/DsD
Dactyl-thoracic index	DTI	FC × 100/TC
Dactyl-costal index	DCI	FC × 100/RW
Relative thickness of the cane bone index	RTCI	FC × 100/HaW
Pelvic index	PI	RW × 100/RL
Longitudinal pelvic index	LPI	RL × 100/HaW
Transversal pelvic index	TPI	RW × 100/HaW
Relative proportionality of the thorax index	RPTI	DsD × 100/HaW

**Table 3:** Descriptive statistics (mean ± standard error) for 173 males and 82 females of Large-sized Podenco Andaluz of Sierra Morena, Cordoba, according to sixteen morphometric variables and eleven indices.

Trait ^1^	All	Males	Females	*p*_Value ^2^
Measurements, cm				
HaW	64.07 ± 0.29 (6.83)	65.27 ± 0.34 (6.43)	61.61 ± 0.42 (5.94)	*p* < 0.001
HR	63.64 ± 0.27 (6.53)	64.69 ± 0.32 (6.18)	61.47 ± 0.42 (5.86)	*p* < 0.001
HsH	38.10 ± 0.18 (7.10)	38.83 ± 0.21 (6.76)	36.59 ± 0.26 (6.05)	*p* < 0.001
BL	67.34 ± 0.28 (6.23)	68.62 ± 0.29 (5.32)	64.73 ± 0.47 (6.25)	*p* < 0.001
DsD	26.67 ± 0.10 (5.66)	26.95 ± 0.13 (6.03)	26.09 ± 0.12 (3.94)	*p* < 0.001
ChW	16.55 ± 0.09 (8.55)	16.81 ± 0.12 (8.74)	16.01 ± 0.13 (7.08)	*p* < 0.001
SL	12.21 ± 0.05 (6.51)	12.35 ± 0.07 (6.58)	11.92 ± 0.08 (5.65)	*p* < 0.001
FL	13.26 ± 0.07 (8.11)	13.39 ± 0.09 (8.64)	12.99 ± 0.10 (6.39)	*p* < 0.01
HL	25.47 ± 0.10 (6.00)	25.73 ± 0.13 (6.08)	24.93 ± 0.15 (5.25)	*p* < 0.001
HW	12.29 ± 0.07 (8.08)	12.55 ± 0.08 (8.10)	11.75 ± 0.08 (5.79)	*p* < 0.001
SW	19.33 ± 0.10 (8.18)	19.63 ± 0.12 (7.64)	18.72 ± 0.18 (8.45)	*p* < 0.001
RW	17.24 ± 0.14 (12.00)	17.60 ± 0.17 (11.98)	16.48 ± 0.20 (10.71)	*p* < 0.001
RL	17.57 ± 0.13 (10.94)	17.94 ± 0.15 (10.30)	16.80 ± 0.21 (11.03)	*p* < 0.001
TC	75.83 ± 0.23 (4.58)	76.45 ± 0.29 (4.67)	74.56 ± 0.33 (3.86)	*p* < 0.001
FC	12.98 ± 0.06 (7.16)	13.25 ± 0.07 (6.37)	12.41 ± 0.10 (6.77)	*p* < 0.001
HC	11.93 ± 0.06 (7.04)	12.12 ± 0.07 (6.93)	11.55 ± 0.08 (6.09)	*p* < 0.001
Indices				
CI	48.35 ± 0.27 (8.42)	48.92 ± 0.36 (9.17)	47.18 ± 0.31 (5.75)	*p* < 0.01
BI	88.89 ± 0.35 (6.04)	89.86 ± 0.41 (5.64)	86.88 ± 0.63 (6.26)	*p* < 0.001
PrI	95.28 ± 0.38 (6.10)	95.20 ± 0.43 (5.56)	95.45 ± 0.78 (7.12)	n.s.
TI	62.13 ± 0.35 (8.55)	62.46 ± 0.45 (8.86)	61.45 ± 0.55 (7.79)	n.s.
DTI	17.13 ± 0.07 (6.71)	17.35 ± 0.08 (6.10)	16.67 ± 0.14 (7.17)	*p* < 0.001
DCI	76.29 ± 0.66 (13.12)	76.29 ± 0.79 (12.81)	76.28 ± 1.22 (13.81)	n.s.
RTCI	20.31 ± 0.10 (7.66)	20.36 ± 0.12 (7.34)	20.20 ± 0.19 (8.30)	n.s.
PI	98.30 ± 0.53 (8.20)	98.28 ± 0.67 (8.43)	98.34 ± 0.88 (7.75)	n.s.
LPI	27.52 ± 0.22 (12.02)	27.56 ± 0.24 (11.02)	27.42 ± 0.44 (13.96)	n.s.
TPI	26.98 ± 0.22 (12.57)	27.03 ± 0.26 (12.05)	26.90 ± 0.42 (13.66)	n.s.
RPTI	41.78 ± 0.21 (7.47)	41.43 ± 0.25 (7.55)	42.49 ± 0.35 (7.05)	*p* < 0.05

^1^ HaW = height at withers; HR = height at rump; HsH = height of substernal hollow; BL = body length; DsD = dorso-sternum diameter; ChW = chest depth; HL = head length; SL = skull length; FL = face length; HW = head width; SW = shoulder width; RW = rump width; RL = rump length; TC = shin circumference; FC = forelimb circumference; HC = hindlimb circumference; CI = cephalic index; BI = body index; PrI = proportionality index; TI = thoracic index; DTI = dactyl-thoracic index; DCI = dactylo-costal index; RTCI = relative thickness of the cane bone index; PI = pelvic index; LPI = longitudinal pelvic index; TPI = transversal pelvic index; RPTI = relative proportionality of the thorax index. ^2^ n.s. = not significantly different.

**Table 4:** Correlation coefficients (males—upper diagonal; females—lower diagonal) between morphometric measures in Large-sized Podenco Andaluz dogs.

Trait ^1^	HaW	HR	BL	DsD	ChW	SL	FL	HL	HW	SW	RW	RL	TC	FC	HC	HsH
HaW		0.90 *	0.58 *	0.22 *	−0.01	0.45 *	0.48 *	0.57 *	0.11	−0.13	0.27 *	0.20 *	0.32 *	0.35 *	0.32 *	0.31 *
HR	0.95 *		0.61 *	0.32 *	0.07	0.42 *	0.39 *	0.50 *	0.09	−0.06	0.28 *	0.15	0.39 *	0.31 *	0.28 *	0.22 *
BL	0.40 *	0.45 *		0.26 *	−0.02	0.42 *	0.31 *	0.47 *	0.06	0.08	0.23 *	0.17 *	0.37 *	0.45 *	0.14	0.12
DsD	0.06	0.08	0.30 *		0.40 *	0.34 *	0.14	0.31 *	−0.01	0.17 *	0.32 *	0.33 *	0.58 *	0.25 *	0.25 *	0.05
ChW	−0.32 *	−0.26 *	−0.09	0.09		−0.05	−0.10	−0.09	0.12	0.32 *	0.20 *	0.14	0.26 *	0.26 *	0.26 *	0.06
SL	0.15	0.13	0.33 *	0.41 *	−0.06		0.26 *	0.87 *	0.03	−0.07	0.33 *	0.42 *	0.51 *	0.28 *	0.26 *	0.14
FL	−0.06	−0.09	0.22	0.19	0.21	0.53 *		0.69 *	0.29 *	0.00	0.32 *	0.37 *	0.28 *	0.37 *	0.37 *	0.15
HL	0.04	0.02	0.33 *	0.37 *	0.07	0.90 *	0.84 *		0.17 *	−0.04	0.41 *	0.51 *	0.52 *	0.39 *	0.37 *	0.17 *
HW	−0.15	−0.10	0.05	0.23 *	0.43 *	0.28 *	0.49 *	0.45 *		0.25 *	0.22 *	0.15	0.03	0.28 *	0.18 *	0.31 *
SW	−0.27 *	−0.25 *	0.13	0.17	0.62 *	0.16	0.38 *	0.29 *	0.41 *		0.40 *	0.23 *	0.21 *	0.25 *	0.09	−0.11
RW	−0.38 *	−0.40 *	−0.20	0.10	0.48 *	0.14	0.35 *	0.27 *	0.34 *	0.42 *		0.72 *	0.35 *	0.25 *	0.20 *	−0.05
RL	−0.34 *	−0.37 *	−0.34 *	0.07	0.39 *	0.13	0.25 *	0.21	0.43 *	0.45 *	0.71 *		0.41 *	0.29 *	0.22 *	−0.08
TC	0.09	0.16	0.32 *	0.32 *	0.30 *	0.44 *	0.30 *	0.42 *	0.36 *	0.55 *	0.20	0.17		0.42 *	0.49 *	−0.01
FC	0.20	0.20	0.29 *	0.41 *	−0.18	0.43 *	0.35 *	0.47 *	0.23 *	−0.04	−0.03	0.11	0.18		0.62 *	0.22 *
HC	0.40 *	0.46 *	0.37 *	0.40 *	−0.13	0.41 *	0.09	0.30 *	0.18	−0.09	−0.15	0.05	0.21	0.76 *		0.23 *
HsH	0.35 *	0.35 *	0.02	−0.33 *	0.03	0.10	0.12	0.12	0.03	0.01	0.18	0.17	0.07	0.11	0.10	

^1^ HaW = height at withers; HR = height at rump; BL = body length; DsD = dorso-sternum diameter; ChW = chest depth; HL = head length; SL = skull length; FL = face length; HW = head width; SW = shoulder width; RW = rump width; RL = rump length; TC = shin circumference; FC = forelimb circumference; HC = hindlimb circumference. * *p* < 0.05.

**Table 5:** Descriptive statistics (mean ± standard error) for 245 males and 176 females of four Spanish dog breeds and one crossbreed population using fifteen morphometric variables.

		Breed ^1^						MANOVA ^3^		
Trait ^2^		CAMP	PAT	MV	GA	MAE	CAMP × MAE	Breed (B)	Sex (S)	B × S
HaW	Males	65.27 ± 0.34 ^b^	52.91 ± 0.50 ^c^	72.40 ± 0.33 ^de^	68.40 ± 0.34 ^e^	78.54 ± 0.55 ^a^	69.18 ± 0.48 ^e^	*p* < 0.001	*p* < 0.001	n.s.
	Females	61.61 ± 0.42 ^e^	49.69 ± 0.52 ^b^	68.11 ± 0.57 ^c^	64.98 ± 0.33 ^d^	74.14 ± 0.51 ^a^	64.50 ± 0.65 ^cde^			
HR	Males	64.69 ± 0.32 ^d^	52.26 ± 0.57 ^b^	71.25 ± 0.39 ^c^	66.57 ± 0.39 ^d^	78.85 ± 0.59 ^a^	70.45 ± 0.37 ^c^	*p* < 0.001	*p* < 0.001	n.s.
	Females	61.47 ± 0.42 ^e^	49.05 ± 0.48 ^b^	66.89 ± 0.58 ^c^	64.19 ± 0.34 ^d^	74.34 ± 0.58 ^a^	65.25 ± 0.63 ^cde^			
BL	Males	68.62 ± 0.29 ^e^	56.18 ± 0.56 ^d^	72.32 ± 0.31 ^c^	67.83 ± 0.53 ^e^	86.72 ± 0.86 ^a^	77.09 ± 1.56 ^b^	*p* < 0.001	*p* < 0.001	n.s.
	Females	64.73 ± 0.47 ^e^	52.95 ± 0.44 ^d^	69.09 ± 0.60 ^c^	65.02 ± 0.55 ^e^	82.80 ± 0.78 ^a^	76.50 ± 0.65 ^b^			
DsD	Males	26.95 ± 0.13 ^b^	20.84 ± 0.26 ^d^	29.85 ± 0.20 ^e^	25.53 ± 0.23 ^c^	34.20 ± 0.49 ^a^	30.91 ± 0.48 ^e^	*p* < 0.001	*p* < 0.001	n.s.
	Females	26.09 ± 0.12 ^e^	19.70 ± 0.24 ^c^	28.00 ± 0.36 ^d^	23.71 ± 0.24 ^b^	33.29 ± 0.37 ^a^	28.50 ± 0.29 ^de^			
ChW	Males	16.81 ± 0.12 ^c^	14.17 ± 0.22 ^d^	17.86 ± 0.12 ^bc^	14.09 ± 0.25 ^d^	25.05 ± 1.84 ^a^	20.36 ± 0.49 ^b^	*p* < 0.001	*p* < 0.01	n.s.
	Females	16.01 ± 0.13 ^c^	13.94 ± 0.25 ^b^	17.15 ± 0.21 ^c^	13.24 ± 0.26 ^b^	21.92 ± 0.49 ^a^	18.50 ± 0.29 ^c^			
SL	Males	12.35 ± 0.07 ^d^	11.21 ± 0.12 ^b^	11.32 ± 0.08 ^c^	13.59 ± 0.15 ^d^	17.38 ± 0.20 ^a^	13.36 ± 0.20 ^c^	*p* < 0.001	*p* < 0.001	n.s.
	Females	11.92 ± 0.08 ^d^	10.39 ± 0.11 ^b^	10.87 ± 0.13 ^c^	12.81 ± 0.14 ^d^	16.23 ± 0.17 ^a^	12.75 ± 0.48 ^c^			
FL	Males	13.39 ± 0.09 ^b^	9.51 ± 0.10 ^d^	14.75 ± 0.10 ^c^	10.57 ± 0.14 ^e^	10.66 ± 0.20 ^e^	15.18 ± 0.33 ^a^	*p* < 0.001	*p* < 0.001	n.s.
	Females	12.99 ± 0.10 ^b^	8.93 ± 0.10 ^a^	13.92 ± 0.15 ^c^	9.89 ± 0.14 ^d^	10.26 ± 0.21 ^cd^	14.50 ± 0.50 ^b^			
HL	Males	25.73 ± 0.13 ^d^	20.58 ± 0.23 ^b^	26.24 ± 0.12 ^d^	23.58 ± 0.15 ^a^	28.09 ± 0.31 ^c^	28.55 ± 0.45 ^c^	*p* < 0.001	*p* < 0.001	n.s.
	Females	24.93 ± 0.15 ^de^	18.84 ± 0.16 ^b^	24.94 ± 0.20 ^e^	22.31 ± 0.16 ^a^	26.43 ± 0.38 ^c^	27.25 ± 0.48 ^cd^			
HW	Males	12.55 ± 0.08 ^b^	10.69 ± 0.09 ^c^	12.82 ± 0.07 ^b^	10.55 ± 0.11 ^c^	14.79 ± 0.13 ^a^	14.73 ± 0.24 ^a^	*p* < 0.001	*p* < 0.001	n.s.
	Females	11.75 ± 0.08 ^b^	9.76 ± 0.07 ^c^	12.03 ± 0.11 ^b^	10.00 ± 0.09 ^c^	13.94 ± 0.21 ^a^	13.25 ± 0.48 ^a^			
SW	Males	19.63 ± 0.12	14.06 ± 0.23	15.70 ± 0.16	11.97 ± 0.13	19.23 ± 0.42	23.73 ± 0.59	n.s.	n.s.	n.s.
	Females	18.72 ± 0.18 ^d^	12.93 ± 0.23 ^b^	15.62 ± 0.31 ^a^	11.38 ± 0.16 ^c^	18.38 ± 0.49 ^d^	20.75 ± 1.25 ^d^			
RW	Males	17.60 ± 0.17 ^a^	12.00 ± 1.56 ^bc^	13.11 ± 0.16 ^c^	9.16 ± 0.22 ^b^	13.63 ± 0.41 ^c^	20.55 ± 0.78 ^a^	*p* < 0.001	n.s.	n.s.
	Females	16.48 ± 0.20 ^b^	10.21 ± 0.18 ^c^	12.91 ± 0.22 ^e^	8.46 ± 0.22 ^d^	13.18 ± 0.57 ^e^	20.25 ± 1.11 ^a^			
RL	Males	17.94 ± 0.15 ^c^	15.48 ± 0.15 ^d^	19.65 ± 0.12 ^b^	16.59 ± 0.21 ^cd^	22.51 ± 0.39 ^a^	22.00 ± 0.59 ^ab^	*p* < 0.001	*p* < 0.01	n.s.
	Females	16.80 ± 0.21 ^d^	14.20 ± 0.21 ^f^	18.83 ± 0.19 ^c^	15.23 ± 0.17 ^e^	20.77 ± 0.34 ^b^	24.50 ± 1.85 ^a^			
TC	Males	76.45 ± 0.29 ^e^	62.96 ± 0.45 ^d^	79.52 ± 0.39 ^e^	70.21 ± 0.57 ^c^	103.23 ± 1.63 ^a^	88.27 ± 1.20 ^b^	*p* < 0.001	*p* < 0.001	*p* < 0.05
	Females	74.56 ± 0.33 ^d^	60.61 ± 0.54 ^c^	76.02 ± 0.74 ^d^	66.93 ± 0.45 ^b^	94.89 ± 1.35 ^a^	78.50 ± 2.90 ^d^			
FC	Males	13.25 ± 0.07 ^c^	10.26 ± 0.13 ^d^	13.18 ± 0.09 ^c^	9.75 ± 0.11 ^d^	16.91 ± 0.21 ^a^	15.91 ± 0.39 ^b^	*p* < 0.001	*p* < 0.001	*p* < 0.05
	Females	12.41 ± 0.10 ^c^	9.30 ± 0.11 ^d^	12.34 ± 0.14 ^c^	9.19 ± 0.14 ^d^	15.45 ± 0.17 ^a^	14.00 ± 0.41 ^b^			
HC	Males	12.12 ± 0.07 ^c^	10.89 ± 1.19 ^bc^	12.29 ± 0.08 ^c^	9.17 ± 0.09 ^b^	16.11 ± 0.21 ^a^	13.45 ± 0.21 ^abc^	*p* < 0.001	*p* < 0.01	n.s.
	Females	11.55 ± 0.08 ^c^	8.84 ± 0.13 ^b^	11.59 ± 0.15 ^c^	8.69 ± 0.13 ^b^	14.74 ± 0.15 ^a^	11.75 ± 0.25 ^c^			

^1^ PAT = Podenco Paternino; MV = Valdueza; GA = Spanish Greyhound; MAE = Spanish Mastiff; CAMP × MAE = Podenco Campanero × Spanish Mastiff; ^2^ HaW = height at withers; HR = height at rump; BL = body length; DsD = dorso-sternum diameter; ChW = chest depth; HL = head length; SL = skull length; FL = face length; HW = head width; SW = shoulder width; RW = rump width; RL = rump length; TC = shin circumference; FC = forelimb circumference; HC = hindlimb circumference; ^3^ n.s. = not significant differences at *p* < 0.05 level. The letters indicate if the means are significantly different or not.

**Table 6:** Discriminant functions for 399 males and 251 females of five Spanish dog breeds and one crossbreed population using fifteen morphometric variables.

Traits ^1^	Wilks’ Lambda	Partial Lambda	F-Remove	*p*-Value ^2^	Toler.	1-Toler.
Males						
FC	0.01	0.69	34.40	*p* < 0.001	0.67	0.33
HaW	0.01	0.84	14.20	*p* < 0.001	0.20	0.80
FL	0.02	0.49	79.01	*p* < 0.001	0.85	0.15
BL	0.01	0.89	9.59	*p* < 0.001	0.64	0.36
DsD	0.01	0.86	12.21	*p* < 0.001	0.69	0.31
TC	0.01	0.80	18.74	*p* < 0.001	0.41	0.59
RL	0.01	0.91	7.46	*p* < 0.001	0.22	0.78
HW	0.01	0.89	9.79	*p* < 0.001	0.90	0.10
RW	0.01	0.93	6.17	*p* < 0.001	0.95	0.05
SW	0.01	0.94	5.11	*p* < 0.001	0.19	0.81
HR	0.01	0.98	1.78	n.s.	0.21	0.79
HC	0.01	0.98	1.62	n.s.	0.86	0.14
SL	0.01	0.99	1.05	n.s.	0.77	0.23
Females						
DsD	0.00	0.86	7.35	*p* < 0.001	0.82	0.18
RW	0.00	0.74	15.85	*p* < 0.001	0.62	0.38
HaW	0.00	0.94	2.92	*p* < 0.05	0.27	0.73
HC	0.00	0.82	9.92	*p* < 0.001	0.42	0.58
FL	0.00	0.84	8.94	*p* < 0.001	0.75	0.25
BL	0.00	0.84	8.96	*p* < 0.001	0.80	0.20
RL	0.00	0.74	16.12	*p* < 0.001	0.78	0.22
ChW	0.00	0.83	9.61	*p* < 0.001	0.72	0.28
SW	0.00	0.83	9.25	*p* < 0.001	0.62	0.38
SL	0.00	0.80	11.28	*p* < 0.001	0.61	0.39
TC	0.00	0.85	8.28	*p* < 0.001	0.64	0.36
HW	0.00	0.88	6.15	*p* < 0.001	0.85	0.15
FC	0.00	0.90	5.28	*p* < 0.001	0.45	0.55
HL	0.00	0.93	3.42	*p* < 0.01	0.51	0.49
RW	0.00	0.95	2.27	*p* < 0.05	0.26	0.74

^1^ HaW = height at withers; HR = height at rump; BL = body length; DsD = dorso-sternum diameter; ChW = chest depth; HL = head length; SL = skull length; FL = face length; HW = head width; SW = shoulder width; RW = rump width; RL = rump length; TC = shin circumference; FC = forelimb circumference; HC = hindlimb circumference. ^2^ n.s. = not significantly different.

**Table 7:** Classification (percentage of accuracy ratio) for 399 males and 251 females of five Spanish dog breeds and one crossbreed population by fifteen morphometric variables.

Breed ^1^	Sex	Percentage	CAMP	CAMP × MAE	MV	GA	MAE	PAT
CAMP	Males (154)	97.40	150	3	1			
	Females (75)	100.00	75					
CAMP × MAE	Males (11)	81.82	2	9				
	Females (4)	100.00		4				
MV	Males (81)	95.06	3		77	1		
	Females (22)	100.00			22			
GA	Males (43)	100.00				43		
	Females (42)	100.00				42		
MAE	Males (39)	87.18		2	3		34	
	Females (34)	100.00					34	
PAT	Males (71)	100.00						71
	Females (74)	100.00						74
Total	Males (399)	96.24	155	14	81	44	34	71
	Females (251)	100.00	75	4	22	42	34	74

^1^ CAMP = *Podenco Campanero*; CAMP × MAE = *Podenco Campanero* × Spanish Mastiff; MV = *Valdueza*; GA = Spanish Greyhound; MAE = Spanish Mastiff; PAT = *Podenco Paternino*.

## Data Availability

The original contributions presented in this study are included in the article. Further inquiries can be directed to the corresponding author(s).
